# Higher Body Mass Index Shows No Evidence of Association with Histopathologic Markers of Aggressiveness in Early-Stage Papillary Thyroid Carcinoma

**DOI:** 10.3390/biomedicines13071681

**Published:** 2025-07-09

**Authors:** Aliki Economides, Demetris Lamnisos, Paris Vogazianos, Konstantinos Giannakou, Savvas Frangos, Vasilis Constantinides, Panagiotis Papageorgis, Panayiotis A. Economides

**Affiliations:** 1 Department of Health Sciences, European University Cyprus, Nicosia 2404, Cyprus; aeconomi@cytanet.com.cy (A.E.); d.lamnisos@euc.ac.cy (D.L.); k.giannakou@euc.ac.cy (K.G.); 2 Economides Thyroid & Endocrinology Center, Engomi 2406, Cyprus; 3 Department of Social and Behavioral Sciences, European University Cyprus, Nicosia 2404, Cyprus; p.vogazianos@euc.ac.cy; 4 Nuclear Medicine Department and Thyroid Cancer Clinic, Bank of Cyprus Oncology Center, Nicosia 2404, Cyprus; savvas.frangos@bococ.org.cy; 5 Department of Endocrine Surgery, Evangelistria Medical Center, Engomi 1095, Cyprus; vascons@doctors.org.uk; 6 Department of Life Sciences, European University Cyprus, Nicosia 2404, Cyprus; p.papageorgis@euc.ac.cy; 7 Department of Medicine, School of Medicine, European University Cyprus, Nicosia 2404, Cyprus

**Keywords:** papillary thyroid carcinoma, obesity, BMI, tumor aggressiveness, thyroid surgery, lymph node metastasis

## Abstract

**Background:** Obesity has been implicated in the pathogenesis and progression of several malignancies, including papillary thyroid carcinoma (PTC), but its role in tumor aggressiveness remains controversial. This study aimed to investigate the association between adiposity, as measured by body mass index (BMI), and histopathological features of aggressiveness in patients with PTC. **Methods:** This single-center retrospective study included 298 consecutive adult patients diagnosed with PTC between 2016 and 2021 at an endocrine referral center. Patients were stratified based on BMI into normal weight (<25 kg/m^2^) and overweight/obese (≥25 kg/m^2^) groups. Clinical, metabolic, and histopathological data were compared between the two groups. **Results:** Overweight/obese patients had significantly higher rates of hypertension, type 2 diabetes, fasting glucose, and triglycerides, as well as lower high-density lipoprotein cholesterol (all *p* < 0.01). Tumor size was similar between groups, with over 85% of tumors measuring ≤ 1 cm (microcarcinomas) and no significant difference in the proportion of tumors > 1 cm (*p* = 0.582). There were no significant differences in multifocality (*p* = 0.269) or extrathyroidal extension (ETE) (*p* = 0.826). Lymph node metastases occurred in 34% of normal weight and 28% of overweight/obese patients, without a statistically significant difference (*p* = 0.402). Lymph node compartment involvement did not significantly differ between groups (*p* = 0.160). **Conclusions:** Despite being associated with adverse metabolic profiles, higher BMI was not linked to tumor aggressiveness in patients with predominantly early-stage PTC. As the incidence of obesity and PTC continues to rise, these findings highlight the need for further research into early-stage PTC biology and more precise risk measures of adiposity beyond BMI alone.

## 1. Introduction

The incidence of thyroid cancer (TC) has sharply risen over recent decades, and it is now the most common endocrine malignancy, representing 3.8% of all new cancer cases globally [1,2]. The predominant histological subtype is papillary thyroid carcinoma (PTC), accounting for 80–85% of well-differentiated TCs [3,4]. In 2020, 586,000 new cases of TC were reported worldwide, while in 2022, there were over 43,000 new cases in the United States, disproportionately affecting women [1,2]. Although PTC is generally indolent with excellent survival, cervical lymph node metastasis is common, affecting up to 90% of patients, and this contributes to the risk of recurrence [5,6]. A thorough preoperative assessment with high-resolution ultrasound is essential for optimizing surgical planning and minimizing the need for reoperation [7].

There has also been an increase in the incidence of overweight and obese individuals, defined by the World Health Organization as a body mass index (BMI) over 25 kg/m^2^, which has paralleled the increase in TC incidence [8]. Elevated BMI is a major modifiable risk factor for multiple cancers, including TC. Additionally, obesity-related factors such as insulin resistance, hormonal imbalances, systemic inflammation, and microbiome alterations may contribute to tumor development and progression, although the underlying mechanisms remain unclear [8].

Obesity is positively associated with an increased risk of TC. Schmid et al. [9] reported that each 5-unit increase in BMI and 0.1-unit increase in waist-to-hip ratio were linked to 30% and 14% higher risks of TC, respectively. A large multinational prospective study by Kitahara et al. [10] also found that the incidence of TC was positively associated with various anthropometric factors, such as height, baseline BMI, waist circumference, BMI during young adulthood, and BMI gain in adulthood. Similarly, Kim et al. [11] demonstrated that young Korean adults with BMI ≥ 25 had a significantly higher risk of developing PTC compared to those with BMI < 23. Moreover, in a study of over 450,000 adults aged 50–71 in the United States, Kitahara et al. [12] confirmed a higher risk of PTC among overweight and obese individuals versus those with normal weight. He et al. [13] also found a positive association between BMI and the risk of differentiated thyroid cancer (DTC), except in men over 50. Lastly, Abiri et al. [14] was also able to confirm a strong and consistent relationship between adiposity and the risk of TC after consolidating data from 27 studies across diverse populations.

However, Matrone et al. [15] reported no significant association between BMI and DTC aggressiveness at diagnosis or during follow-up, concluding that BMI should not influence postsurgical management. A study on UK Biobank data by Fussey et al. [16] found no evidence supporting a causal relationship between obesity and either benign nodular disease or TC. In a cohort of 641 patients with thyroid nodules, Rotondi et al. [17] similarly observed no meaningful correlation between obesity and DTC. Ahmadi et al. [18] also concluded that BMI does not improve the prediction of cancer risk during thyroid nodule assessment.

Kim et al. [19] and Wang et al. [20] found that elevated BMI was associated with larger tumor size, multifocality, extrathyroidal extension, lymph node metastasis, and advanced TNM staging, thereby highlighting obesity as a clinical marker of more aggressive disease. Furthermore, sex-specific effects were found by Huang et al. [21], with obese males having more aggressive tumor features. Meanwhile, Lee et al. [22] identified a link between obesity and BRAFV600E mutation, suggesting a biological pathway behind the influence of obesity on tumor behavior. In addition, Li et al. [23] found that reduced adiponectin levels, which are common in obese patients, were associated with characteristics of aggressive PTC. Di Filippo et al. [24] and Matrone et al. [15] reported that overweight and obese individuals were more likely to exhibit high-risk histopathological subtypes and less favorable treatment outcomes. However, Mele et al. [25] cautioned against overreliance on BMI alone, recommending a comprehensive body composition analysis (i.e., bioelectrical impedance or DEXA scans) for better risk assessment. Moreover, Li et al. [26] identified a significant link between obesity and tumor calcification, a marker of invasive behavior. Lastly, Zhang et al. [27] found no significant association between BMI and advanced TNM stage or BRAFV600E mutation, although they did report increased multifocality and bilaterality among obese patients.

Our previous systematic review and meta-analysis revealed an association between elevated BMI and aggressive clinicopathological features of PTC, including larger tumor size, multifocality, and lymph node metastasis [28]. Moreover, we also found an association between adiposity and increased malignancy risk, even in specific subgroups such as patients with Hashimoto’s thyroiditis [29,30]. This study evaluates the link between elevated BMI and more invasive disease features, such as tumor size, multifocality, extrathyroidal extension, and lymph node metastasis.

## 2. Materials and Methods

### 2.1. Patients and Data Recording

This retrospective study included 298 consecutive patients diagnosed with PTC from 2016 to 2021 who underwent lobectomy or total thyroidectomy, excluding pediatric patients. All patients completed their medical and endocrine evaluations at the Thyroid and Endocrinology Center, Engomi 2406, Cyprus, a referral clinic and a teaching affiliate of the European University Cyprus School of Medicine. BMI was calculated based on the weight and height of the patient during the initial evaluation. Patients were stratified into two groups based on BMI: normal (<25 kg/m^2^) and overweight/obese (≥25 kg/m^2^). We retrospectively reviewed medical records and pathology reports for all included patients. Cases with incomplete histopathological data were excluded from the analysis. Patients underwent thyroidectomy based on preoperative FNA cytology. Experienced thyroid surgeons performed all surgeries. In most cases, central lymph node (LN) dissection was routinely conducted, with lateral LN dissection performed when lateral LN involvement was suspected by US or diagnosed preoperatively by FNA. Surgical specimens were examined by experienced pathologists following standard histopathological protocols. The study population was homogeneous, comprising almost entirely ethnic Greek Cypriot individuals. The Cyprus National Bioethics Committee approved this study (ΕΕΒΚ ΕΠ 2020.01.38). Patients’ personal information and identities were kept confidential throughout the study.

### 2.2. Statistical Analysis

Numerical variables were first assessed for normality using the Shapiro–Wilk test. Normally distributed variables are presented as mean ± SD and compared via an independent-sample *t*-test. Nonnormally distributed variables are summarized as medians (interquartile range) and compared via the Mann–Whitney *U*-test.

Categorical variables are reported as counts and percentages. Inter-group comparisons employ Pearson’s χ^2^ test when all expected cell counts are ≥5. If any expected count in a 2 × 2 table falls below 5, Fisher’s exact test is used. For larger RxC tables with low counts, the Freeman–Halton extension of Fisher’s exact test is applied.

To obtain adjusted *p*-values, numerical outcomes were modeled via multiple linear regression with BMI group, gender, and age as independent variables. The *t*-test for the BMI group coefficient was reported. Binary outcomes were modeled via logistic regression, with significance assessed via a likelihood ratio χ^2^ comparison of full (BMI group + gender + age) versus reduced (gender + age) models. Ordinal and nominal outcomes were analyzed via proportional odds regression and multinomial logistic regression, respectively, and both were compared to a model omitting the BMI group to derive a single adjusted *p*-value.

All statistical analyses were performed in R version 4.2.0 (R Foundation for Statistical Computing, Vienna, Austria), and two-sided *p* < 0.05 was considered statistically significant.

## 3. Results

### Patient Characteristics

Among 298 patients with PTC, 123 (41.3%) had BMI < 25 kg/m^2^, while 175 (58.7%) had BMI ≥ 25 kg/m^2^. Overweight/obese patients were significantly older (mean age: 46.3 vs. 42.3 years; adjusted *p* = 0.021) and had higher systolic and diastolic blood pressure (SBP: 123.4 vs. 115.1 mmHg, adjusted *p* = 0.002; DBP: 76.0 vs. 72.0 mmHg, adjusted *p* = 0.002), as well as higher fasting glucose and triglycerides (both adjusted *p* < 0.001). High-density lipoprotein cholesterol was significantly lower in the overweight/obese group (adjusted *p* < 0.002). No statistically significant differences were observed in terms of thyroid-stimulating hormone (TSH) levels. Patients in the overweight/obese group had significantly higher rates of hypertension and type 2 diabetes (both adjusted *p* < 0.001).

Total thyroidectomy was the most frequently performed procedure across both groups. Central lymph node dissection was performed in 78.2% of the normal weight group and 76.6% of the overweight/obese group (adjusted *p* = 0.792), with similar rates of lateral neck dissection (adjusted *p* = 0.085). There were no significant differences in mean tumor size between the normal weight versus overweight/obese groups (7.6 vs. 7.9 mm, *p* = 0.568). Most tumors were microcarcinomas (≤1 cm), observed in 105 patients (83.3%) in the normal weight group and in 145 patients (84.3%) in the overweight/obese group, while larger tumors (>1 cm) were present in 21 (16.7%) and 27 (15.7%) patients, respectively, with no significant difference between groups (adjusted *p* = 0.582).

There were no significant differences between groups in multifocality (adjusted *p* = 0.269), bilaterality (adjusted *p* = 0.268), or extrathyroidal extension (adjusted *p* = 0.826). Histologically confirmed lymph node metastases were seen in 34% of normal weight and 28% of overweight/obese patients (adjusted *p* = 0.402). There were no significant differences in lymph node compartment involvement (adjusted *p* = 0.160). All results are presented in Table 1 and Table 2.

We performed an analysis using three BMI categories (normal weight, overweight, and obese), as well as modeling BMI as a continuous variable, both of which yielded similar results. 

## 4. Discussion

This study examined the association of elevated BMI with tumor aggressiveness in patients with PTC. Although overweight/obese individuals had significantly more adverse metabolic features (i.e., hypertension, type 2 diabetes, and dyslipidemia), BMI itself was not linked to features of tumor aggressiveness (tumor size, multifocality, bilaterality, extrathyroidal extension, vascular invasion, or lymph node (LN) metastasis). In contrast, previous studies have suggested a positive association between obesity and aggressive PTC characteristics, such as larger tumor size, increased extrathyroidal extension, and higher rates of LN metastasis or BRAFV600E mutations [19,20,21]. However, our findings agree with other studies that found no such relationship. For instance, Matrone et al. [15], Rotondi et al. [17], and Ahmadi et al. [18] concluded that BMI does not independently predict TC behavior. Fussey et al. [16] further demonstrated no causal link between obesity and TC using Mendelian randomization.

Obesity is associated with multiple oncogenic mechanisms, such as chronic low-grade inflammation, hormonal imbalance, adipokine dysregulation, and altered TSH dynamics. Adipokines, such as adiponectin, exert anti-inflammatory and anti-proliferative effects, and their reduction in obese individuals may promote PTC progression through autophagy-related pathways [23]. However, the elevated leptin levels in obesity can activate protumorigenic signaling pathways (JAK/STAT, MAPK, PI3K/Akt), thereby enhancing thyroid cell proliferation and invasion [31]. While elevated TSH has been implicated in thyroid follicular cell proliferation and nodule formation in prior studies, our findings suggest that other obesity-related mechanisms may also contribute. Specifically, the excess estrogen, insulin resistance, and elevated IGF-1 levels commonly observed in obesity may promote tumor growth and angiogenesis [31].

Previous studies argue that BMI has limitations as a standalone metric and that various metabolic obesity phenotypes must also be considered. Kwon et al. [32] reported that obesity increased the risk of TC in males regardless of metabolic status, whereas in females, only those with metabolically unhealthy obesity had an increased risk. This was confirmed by Hedayati et al. [33], showing that metabolic dysfunction, rather than just elevated BMI, was consistently associated with a higher risk of TC in both males and females. Similarly, Pasqual et al. [34] found that BMI, central obesity, and metabolic conditions (i.e., metabolic syndrome and polycystic ovary syndrome) were all independently linked to TC in females, even after adjusting for BMI. Maleki et al. [35] identified obesity as a leading contributor to the increasing TC incidence worldwide, alongside smoking and dietary factors. Likewise, Islami et al. [36] estimated that 7.6% cases of invasive cancer and 44% of cancer deaths were associated with excess body weight in the United States in 2019, ranking just behind tobacco use in cancer burden.

This study has several limitations. First, the retrospective study design limits causal inference and may introduce residual confounding. Second, BMI was the only measure of adiposity used in this study, whereas other factors that can better predict tumor risk, such as fat distribution, visceral adiposity, and metabolic obesity phenotypes, were not assessed. Third, data regarding the duration of obesity, insulin resistance, dietary patterns, and physical activity were unavailable. Lastly, most tumors in our cohort were microcarcinomas, predominantly reflecting early-stage disease. This limits the variability in aggressive pathological features, potentially obscuring detectable differences between the two groups. However, this also represents a unique strength of our study, since analyzing early-stage tumors can provide information on the initial impact of adiposity on tumor biology before progression occurs. This is particularly relevant considering the rising incidence of TC and widespread ultrasound screening, which detects cancers at early stages. Although our study examined a well-characterized cohort with detailed clinicopathologic data, the sample size may have restricted our ability to detect subtle differences in tumor aggressiveness. Future studies with stratified analyses by tumor size are warranted to assess whether adiposity has a more pronounced effect in larger tumors.

Future research should involve molecular analyses to better understand the biological mechanisms linking adiposity to tumor aggressiveness in PTC. Prospective studies should utilize immunohistochemistry (IHC) or Western blot techniques to identify relevant molecular markers, which could help validate the histopathologic findings and provide deeper insight into the biological mechanisms at play. Next-generation IHC using molecular markers such as B-Raf proto-oncogene, serine/threonine kinase (BRAF) V600E, rat sarcoma viral oncogene homolog (RAS) Q61R, pan-tropomyosin receptor kinase (pan-TRK), anaplastic lymphoma kinase (ALK), phosphatase and tensin homolog (PTEN), and beta-catenin shows great promise for the diagnosis and molecular classification of thyroid carcinomas [37].

In conclusion, although higher BMI is associated with metabolic comorbidities, our current data do not support an association with aggressive PTC features. These results add to growing evidence that BMI alone may not be a reliable prognostic marker in TC. Given the mixed findings in the literature, further research is warranted. Future studies with a prospective, multicenter design should include more comprehensive adiposity assessments, such as waist circumference, visceral fat, bioimpedance analysis, metabolic parameters, and molecular tumor profiling.

## Figures and Tables

**Table 1:** Demographic, clinical, and metabolic characteristics stratified by body mass index (<25 vs. ≥25) (n = 298).

Variable	Subcategory/Statistic	BMI < 25	BMI ≥ 25	*p*-Value †	Adjusted *p*-Values §
Gender	Male	12 (20.0%)	48 (80.0%)		
	Female	114 (47.9%)	124 (52.1%)		
	Total	126 (42.3%)	172 (57.7%)	<0.001	<0.001
Age (years)	Mean (SD)/Median (IQR)	42.3 (12.4)/41.0 (19.5)	46.3 (12.4)/47.0 (16.8)	0.005	0.021
SBP (mmHg)	Mean (SD)/Median (IQR)	115.1 (14.1)/113.5 (20.3)	123.4 (15.8)/120.0 (20.0)	<0.001	0.002
DBP (mmHg)	Mean (SD)/Median (IQR)	72.0 (7.4)/72.0 (9.3)	76.0 (8.6)/75.0 (12.0)	<0.001	0.002
TSH (mIU/L)	Mean (SD)/Median (IQR)	1.85 (1.40)/1.50 (1.56)	1.87 (1.42)/1.70 (1.45)	0.604	0.556
Fasting Glucose (mg/dL)	Mean (SD)/Median (IQR)	90.0 (8.4)/90.0 (12.4)	97.9 (16.8)/95.0 (18.0)	<0.001	0.002
Total Cholesterol (mg/dL)	Mean (SD)/Median (IQR)	187.6 (40.3)/190.3 (45.2)	192.4 (43.7)/190.5 (59.1)	0.371	0.630
HDL (mg/dL)	Mean (SD)/Median (IQR)	60.9 (14.7)/59.0 (18.2)	51.6 (13.0)/51.9 (16.0)	<0.001	<0.002
LDL (mg/dL)	Mean (SD)/Median (IQR)	111.7 (35.6)/105.6 (45.0)	124.2 (45.5)/116.7 (54.0)	0.051	0.072
Triglycerides (mg/dL)	Mean (SD)/Median (IQR)	75.6 (41.2)/65.5 (40.9)	104.1 (53.7)/94.0 (60.8)	<0.001	<0.001

**Abbreviations:** BMI, body mass index; SBP, systolic blood pressure; DBP, diastolic blood pressure; TSH, thyroid-stimulating hormone; HDL, high-density lipoprotein; LDL, low-density lipoprotein; IQR, interquartile range; SD, standard deviation. † *p*-values calculated using the chi-square test or Mann–Whitney U-test, as appropriate. § Adjusted *p*-values derived from regression models controlling for age and gender. See Statistical Analysis section for full details.

**Table 2:** Histopathological features stratified by body mass index (<25 vs. ≥25).

Variable	BMI < 25 (n, %)	BMI ≥ 25 (n, %)	*p*-Value †	Adjusted *p*-Value (§)
Histology			1	0.700
Classic PTC	113 (42.2%)	155 (57.8%)		
Follicular PTC	13 (43.3%)	17 (56.7%)		
Multifocal PTC			0.308	0.268
- No	97 (40.8%)	141 (59.2%)		
- Yes	29 (48.3%)	31 (51.7%)		
Bilateral PTC			0.306	0.268
- No	106 (41.1%)	152 (58.9%)		
- Yes	20 (50.0%)	20 (50.0%)		
Tumor Size, cm			0.874	0.582
- ≤1 cm	105 (42.0%)	145 (58.0%)		
- >1 cm	21 (43.8%)	27 (56.2%)		
ETE			1.000	0.826
- No	97 (42.2%)	133 (57.8%)		
- Yes	29 (42.6%)	39 (57.4%)		
Vascular Invasion			1.000 ‡	0.529
- No	124 (42.2%)	170 (57.8%)		
- Yes	2 (50.0%)	2 (50.0%)		
LN Metastasis			0.312	0.402
- No	83 (40.3%)	123 (59.7%)		
- Yes	43 (46.7%)	49 (53.3%)		
Location of LN Metastasis			0.311 ‡	0.160
- No	83 (40.3%)	123 (59.7%)		
- Central only	24 (43.6%)	31 (56.4%)		
- Central and lateral	12 (44.4%)	15 (55.6%)		
- Lateral only	7 (70.0%)	3 (30.0%)		

**Abbreviations:** BMI, body mass index; PTC, papillary thyroid carcinoma; ETE, extrathyroidal extension; LN, lymph node. † *p*-value from Pearson’s chi-square test unless indicated. ‡ Fisher’s exact test is used due to expected cell count < 5. § Adjusted *p*-values derived from regression models controlling for age and gender. See Statistical Analysis section for full details.

## Data Availability

The data supporting this study’s findings are available from the corresponding author upon reasonable request and with appropriate ethical clearance.

## Abbreviations

The following abbreviations are used in this manuscript:

BMI	Body mass index
DBP	Diastolic blood pressure
DOI	Digital object identifier
DTC	Differentiated thyroid cancer
ETE	Extrathyroidal extension
HDL	High-density lipoprotein
IQR	Interquartile range
LDL	Low-density lipoprotein
LN	Lymph node
PTC	Papillary thyroid carcinoma
SBP	Systolic blood pressure
SD	Standard deviation
TC	Thyroid cancer
TSH	Thyroid-stimulating hormone
